# Are Increases in Purchasing Alcohol‐Free and Low‐Alcohol Drinks Associated With Attempts to Cut Down on Alcohol Consumption in England? A Time Series Analysis

**DOI:** 10.1111/dar.70278

**Published:** 2026-10-09

**Authors:** Abigail K. Stevely, Inge Kersbergen, Jamie Brown, Ellen McGrane, Sarah Jackson, Luke B. Wilson, John Holmes

**Affiliations:** ^1^ Sheffield Addictions Research Group School of Medicine and Population Health, University of Sheffield Sheffield UK; ^2^ Department of Psychology University of Bath Bath UK; ^3^ Department of Behavioural Science and Health University College London London UK; ^4^ SPECTRUM Consortium Edinburgh UK

**Keywords:** alcohol drinking, harmful drinking, purchasing, time series analysis, zero‐alcohol

## Abstract

**Introduction:**

Alcohol‐free and low‐alcohol products (≤ 1.2% ABV; no/lo products) are a growing market in high‐income countries, including Great Britain. These products may support people in reducing alcohol consumption, but their impact on public health is unclear. To address this gap, we analysed whether changes over time in the prevalence of households purchasing no/lo products were associated with at‐risk drinkers reporting: (i) high motivation to cut down; (ii) cut down attempts; (iii) serious cut down attempts; and (iv) using no/lo products during attempts.

**Methods:**

We used time series analysis of continuous household purchasing panel data and monthly cross‐sectional survey data from adults in England (2018–2023). The primary exposure was household prevalence of purchasing no/lo products. Outcomes focused on cut down attempts in the last 12 months amongst at‐risk drinkers, defined as scoring ≥ 5 on the consumption questions or ≥ 8 on all questions of the Alcohol Use Disorders Identification Test.

**Results:**

Changes in household no/lo purchasing were not significantly associated with population‐level prevalence of high motivation to cut down (*B*: −0.001 percentage points [pp]; 95% CI −0.013 to 0.011), current or past 12‐month attempts (*B*: −0.012 pp; 95% CI −0.025 to 0.000), most recent attempt being reported as serious (*B*: 0.004 pp; 95% CI −0.016 to 0.023) or use of no/lo during the most recent attempt (*B*: 0.012 pp; 95% CI −0.023 to 0.046). These narrow confidence intervals do not include clinically significant effect sizes.

**Discussion and Conclusions:**

From 2018 to 2023, increases in the population‐level prevalence of no/lo purchasing in England were not significantly associated with changes in the prevalence of cut down attempts amongst at‐risk drinkers.

## 1 Introduction

Alcohol‐free (up to 0.05% alcohol by volume [ABV]) and low‐alcohol (up to 1.2% ABV) drinks, hereafter no/lo drinks, are beers, ciders, wines or other typically alcoholic drinks that contain little or no alcohol [1, 2]. There is a growing market for these products in many high‐income countries, including in Great Britain where sales have grown rapidly since 2019. However, they remain a small proportion of the overall alcohol market [1]. No/lo drinks made up 0.7% of on‐trade (i.e., pubs and other licensed venues) alcohol sales volume in 2023 following a period of accelerating growth over time [3]. In the off‐trade (i.e., supermarkets and other off‐licences) no/lo drinks made up 1.9% of alcohol sales volume in 2023 with a steady rate of growth over time.

Policy makers and public health actors have argued that these products have potential to reduce alcohol consumption and its associated social, financial and health harms [4, 5]. Conversely, some public health actors have raised concerns about potential negative impacts. For example, marketing of no/lo drinks may indirectly promote standard alcoholic drinks (e.g., brand sharing or surrogate marketing), no/lo products could encroach into alcohol‐free spaces and expose people trying to avoid alcohol consumption to cues to drink and alcohol producers may use promotion of no/lo products to discourage or distract governments from introducing effective alcohol control policies [6, 7, 8, 9, 10].

The evidence base on whether no/lo drinks are associated with reduced alcohol consumption is limited, and several existing studies have been supported directly or indirectly by the alcohol industry. For instance, a study supported by the brewer AB InBev found that purchasing of alcohol‐free products was associated with a small decrease in purchasing of alcohol in Great Britain [11]. Further analyses of the same dataset found the launch or purchasing of a no/lo beer that shares branding with a standard alcoholic beer was associated with reduced purchasing of the standard beer [12]. However, these studies only covered off‐trade purchasing and the data series ended in 2019, before much of the recent growth in no/lo sales and the launch of many leading products [13, 14]. Separately, a controlled experiment found that introducing a draught alcohol‐free beer in bars and pubs in England reduced total sales volume of draught alcoholic beer [15]. A randomised controlled trial funded by the brewer Asahi found that providing large amounts (i.e., up to 216 bottles) of free no/lo beer or cocktails to heavier drinkers for 12 weeks was associated with a reduction in alcohol consumption 8 weeks later [16].

The existing literature has focused on understanding sales and purchasing of no/lo and alcoholic products and has paid less attention to how no/lo products are used by consumers. One potential pathway through which the growth of no/lo products could influence public health is by changing the broader drinking environment. As the market for these products expands, their availability, visibility in retail settings and marketing practices may increase awareness and perceived social acceptability of using them as substitutes for alcoholic drinks [17]. These changes may lower practical or social barriers to reducing alcohol consumption, particularly in contexts where drinking alcohol is normative (e.g., social occasions or licensed venues). In this way, the increasing prominence of no/lo products in the market could make it easier or more appealing for people who drink at risky levels to attempt to reduce their alcohol consumption.

This mechanism operates at the population level rather than necessarily through individual purchasing decisions. Increased availability and uptake of no/lo products in the population may influence drinkers' behaviour even if they are not themselves purchasing these products, for example by increasing awareness of alcohol‐free alternatives, changing perceived norms around drinking or making substitution more feasible in social settings.

There is some evidence that this might be the case, as qualitative interviews with adults in both the United Kingdom and the United States who were trying to reduce their alcohol consumption or who were in recovery from alcohol dependence have shown that these groups largely view no/lo drinks as replacements for alcoholic drinks [18, 19, 20]. There is also some precedent for this mechanism, as the smoking cessation literature shows that nicotine replacement therapy may promote later quit attempts [21, 22]. Evidence on this mechanism has informed National Institute for Health and Clinical Excellence guidelines on smoking [23]. On the other hand, people who have cut down their alcohol consumption, or are attempting to do so, may drink more no/lo drinks as an alternative to soft drinks, rather than as a direct replacement for alcoholic drinks. Therefore, increases in cut down attempts may drive purchasing of no/lo drinks at the population level, rather than the reverse.

If wider sales of no/lo drinks do influence people drinking at risky levels to reduce their consumption, this has implications for policy makers, who may wish to introduce policies supporting the availability and reducing the price of these products. The present study therefore uses time series analysis to evaluate whether changes over time in the population‐level prevalence of households buying no/lo products are associated with changes in the prevalence of people who drink at risky levels: (i) reporting high motivation to cut down; (ii) attempting to cut down alcohol consumption; (iii) making a serious attempt to cut down; and (iv) using no/lo products to help them to cut down alcohol consumption. Evidence that increases in no/lo purchasing are associated with increases in our outcomes would support the hypothesis that these products make it easier and more appealing to make cut down attempts. A lack of association may suggest that no/lo drinks are used as an alternative to soft drinks rather than as a direct substitute for alcoholic drinks.

## 2 Methods

### 2.1 Research Design

Time series analysis of continuous household purchasing panel data collected by Worldpanel by Numerator and individual‐level monthly cross‐sectional survey data from the Alcohol Toolkit Study (ATS), both collected between January 2018 and December 2023. We used Worldpanel data to create our exposure measure and ATS data to create our outcome measures. Open Science Framework pre‐registration: https://osf.io/47a93.

### 2.2 Data

Worldpanel by Numerator (formerly Kantar) is a market research company specialising in panel data. Its Take Home panel collects data from a continuous panel of 30,000 households across Great Britain. Quota samples are based on geographical region, household size and age of main shopper that are representative of households in Great Britain. Households provide data on a continuous basis over time, and attrition is addressed with continuous replenishment. Whilst participating in the study, households use barcode scanners to record off‐trade purchases of no/lo and alcoholic drinks brought into the home. The dataset includes stock‐keeping unit (a sales unit, e.g., a four pack of a particular beer), natural volume, packaging type (e.g., bottles or cans), number of items in multipacks and the ABV. Worldpanel by Numerator calculates 4‐weekly panel weights which we use in all analyses. Since our analyses use monthly data, we used a monthly weighted average of the 4‐weekly weights for each household. Analyses include households in England only.

The Alcohol Toolkit Survey is a monthly repeat cross‐sectional survey of approximately 1700 people (age 16+) in England. The sample is nationally representative and recruits using a mixture of quota and random probability sampling. The published ATS protocol contains further detailed methods [24]. Participants completed the survey face‐to‐face until April 2020, when ATS moved to computer assisted telephone interviews. The ATS includes questions on both consumption of standard alcoholic drinks and no/lo drinks. Participants are also asked about their attitudes towards drinking, motivation to cut down and their attempts to cut down alcohol consumption. We use ATS survey weights in all analyses.

### 2.3 Measures

#### 2.3.1 Exposure

Our exposure measure is the proportion of households present in Worldpanel by Numerator's Take Home panel each month who record purchases of no/lo products in that month.

For sensitivity analyses, we use the same outcome measures but a secondary exposure measure that limits the sample to increasing and higher risk purchasing households, defined as those purchasing more than 14 units (1 UK unit = 8 g or 10 mL of pure alcohol) of alcohol per adult in the household per week on average that month. From 2018 to 2021 Worldpanel by Numerator used a categorical variable for number of adults in the household (1, 2 or 3+). We use random imputation to categorise 3+ households into 3, 4, 5 or 6 adult households to avoid overestimating purchasing per adult in these households. From 2022 onward Worldpanel by Numerator's Take Home panel collected the exact number of adults per household.

#### 2.3.2 Outcomes

Our four outcome measures focus on people who drink at risky levels, as the ATS only collects data on attempts to cut down from this group (defined as scoring ≥ 5 on the consumption questions or scoring ≥ 8 on all questions of the Alcohol Use Disorders Identification Test [AUDIT]). Note that we mistakenly did not mention the ≥ 8 inclusion criteria on the full AUDIT in our pre‐registered protocol. Since the ATS is a repeat cross‐sectional study, this measure excludes respondents who were drinking at risky levels in the past year but successfully cut down and no longer meet the threshold at the time of interview.

Our four outcome measures are:

*High motivation to cut down*: Participants are asked ‘Which of the following best describes you?’ with seven response options (I REALLY want to cut down on drinking alcohol and intend to in the next month; I REALLY want to cut down on drinking alcohol and intend to in the next 3 months; I want to cut down on drinking alcohol and hope to soon; I REALLY want to cut down on drinking alcohol but I don't know when I will; I want to cut down on drinking alcohol but haven't thought about when; I think I should cut down on drinking alcohol but don't really want to; I don't want to cut down on drinking alcohol). We create a binary measure and analyse the proportion who report the three highest motivation options, which indicate intention to cut down drinking soon.

*Current or past 12 month cut down attempts*: The proportion of people who responded ‘Yes’ to either of two questions asking whether they: (i) are currently trying to restrict their alcohol consumption; or (ii) have tried to do so in the last 12 months.

*Most recent cut down attempt was a serious attempt*: The proportion of those who made an attempt to cut down in the last 12 months (including current attempts) who responded ‘Yes’ to the question ‘During your most recent attempt to restrict your alcohol consumption, was it a serious attempt to cut down on your drinking permanently?’

*Used no/lo drinks during most recent cut down attempt*: Participants are asked which methods they used to ‘try to help you cut down’ and no/lo drinks was added as an option to select in October 2020. We measure the proportion of those who made an attempt to cut down in the last 12 months (including current attempts) who selected no/lo drinks. Since the ATS did not include this question in the survey in England in six waves (05/2022, 07/2022, 09/2022, 11/2022, 12/2022 and 07/2023), we used multiple imputation to impute missing values using sociodemographic variables (sex, age, region, social grade, AUDIT questions 1 to 3) as predictors. We used the SPSS command *IMPUTE* with *METHOD = AUTO*.

#### 2.3.3 Controlling for Time‐Varying Confounders

Our analyses include data that spans the COVID‐19 pandemic. The pandemic and associated restrictions substantially affected alcohol sales in England. Lockdown measures included temporary closure of on‐trade venues resulting in effectively no on‐trade sales of standard alcoholic or no/lo drinks and increased sales in the off‐trade.

To control for the COVID‐19 pandemic we include the Oxford COVID‐19 Government Response Tracker (OxCGRT) response rate for England where this improves model fit (based on Bayesian information criterion), which has been previously used in alcohol time series analyses [25, 26]. The OxCGRT provides indexed values indicating the severity of societal restrictions implemented by governments worldwide to hinder COVID‐19 transmission. The OxCGRT systematically collected information on several different common policy responses governments took between 2020 and 2022, recorded these on a scale to reflect the extent of government action and aggregated the resulting scores into a suite of policy indices.

We also include a binary control for pre‐ and post‐COVID‐19 from April 2020 (following the introduction of lockdown measures) where this improves model fit. The pre‐registered analysis plan did not include controlling for a change in long‐term trend, or slope, in April 2020. However, where we identified a possible change in slope on visual inspection of the outcome series and found that the inclusion of a slope change term improves model fit, models include this additional control.

### 2.4 Statistical Analysis

We used controlled SARIMAX models to estimate the associations between the proportion of households purchasing no/lo products and each of our four outcome measures.

We analysed monthly time series and conducted Granger causality tests using the Toda and Yamamoto procedure [27] to test the assumption that there is no reverse causality between lagged values of our outcomes and our exposures. This assumption was supported for our primary analyses and violated for one of our sensitivity analyses. We therefore did not fit the relevant sensitivity SARIMAX model (see Table S1).

To fit SARIMAX models, we used the following model selection process:

- We used visual inspection and augmented Dickey–Fuller tests to identify whether the exposure and outcome series were stationary. We included linear trend terms, applied transformations and differencing as required to produce stationary series.
- We then examined patterns of auto‐correlation in the outcome series using Auto‐Correlation Function plots and Partial Auto‐Correlation Function plots to select an initial SARIMAX model specification.
- Finally, we applied an iterative process of examining model residuals and model fit to select the most appropriate SARIMAX model and assess whether this produces residuals that resemble white noise.

We also used the approach outlined above to conduct sensitivity analyses predicting each of our four outcome series with an alternative exposure series focused on only increasing or higher risk households who report purchases of no/lo products. We conducted an unplanned sensitivity analysis where the first three lags of the exposure variable were included in each model such that changes in the exposure may predict outcomes 1, 2, or 3 months later, none of which were statistically significant.

Time series analyses were conducted using StataNow 18 MP. Key commands used were *dfuller* for augmented Dickey–Fuller tests, *arima* for fitting models, *wntestq* and *corrgram* for white noise tests, and *var* and *test* for Granger causality tests. Multiple imputation was conducted using SPSS. Our alpha level was 0.05.

### 2.5 Ethics Statement

This study was approved by the University of Sheffield's ethics committee. The University College London Ethics Committee granted ethical approval for the ATS (ID 0498/001).

Use of the Worldpanel by Numerator's Take Home dataset is allowed under the terms of the contract and nondisclosure agreement between Worldpanel by Numerator and the University of Sheffield, which requires research outputs to be submitted to the data provider ahead of publication. The data provider's right to request changes is limited to matters of accuracy regarding the data. The data provider played no further role in the research process, including in conception, design, analysis, interpretation, write‐up or the decision to publish. All analysis and interpretation was conducted independently of Worldpanel by Numerator. Worldpanel cannot independently verify the findings, nor can it endorse the views or findings of this report.

## 3 Results

Table 1 presents descriptive statistics on the mean levels of exposure and outcome measures across the study period. Overall, the prevalence of no/lo purchasing was higher amongst households purchasing at increasing or higher risk levels than among all households (4.78% vs. 2.89%).

**TABLE 1:** Mean levels of exposure and outcome measures based on full monthly time series (2018–2023).

Exposure measure	Mean value (%)
Proportion of households who record purchases of alcohol‐free or low‐alcohol products in each month	2.9
Sensitivity analysis: Proportion of increasing or higher risk households^a^ who record purchases of alcohol‐free or low‐alcohol products in each month	4.8

Outcome measures amongst at‐risk drinkers^b^	Mean value (%)
High motivation to cut down	13.2
Current or past 12 month cut down attempts	30.8
Most recent cut down attempt was a serious attempt^d^	38.2
Used alcohol‐free or low‐alcohol drinks during most recent cut down attempt^c^, ^d^	29.0

^a^ Increasing or higher risk households were defined as purchasing more than 14 units (1 UK unit = 8 g or 10 mL pure alcohol) of alcohol per adult in the household per week on average that month.

^b^ At‐risk drinkers were defined as scoring ≥ 5 on the consumption questions or scoring ≥ 8 on all questions of the Alcohol Use Disorders Identification Test (AUDIT‐C screening test).

^c^ Time series starts in October 2020.

^d^ As a proportion of those who made an attempt to cut down in the last 12 months (including current attempts).

The proportion of households recording purchases of no/lo products each month increased across the study period (Figure 1). The rate of increase was faster amongst increasing and higher risk households, although seasonal patterns were similar with higher rates of purchasing in the summer and at the end of each year. The extent of seasonal variation increased between 2018 and 2023.

**FIGURE 1:**
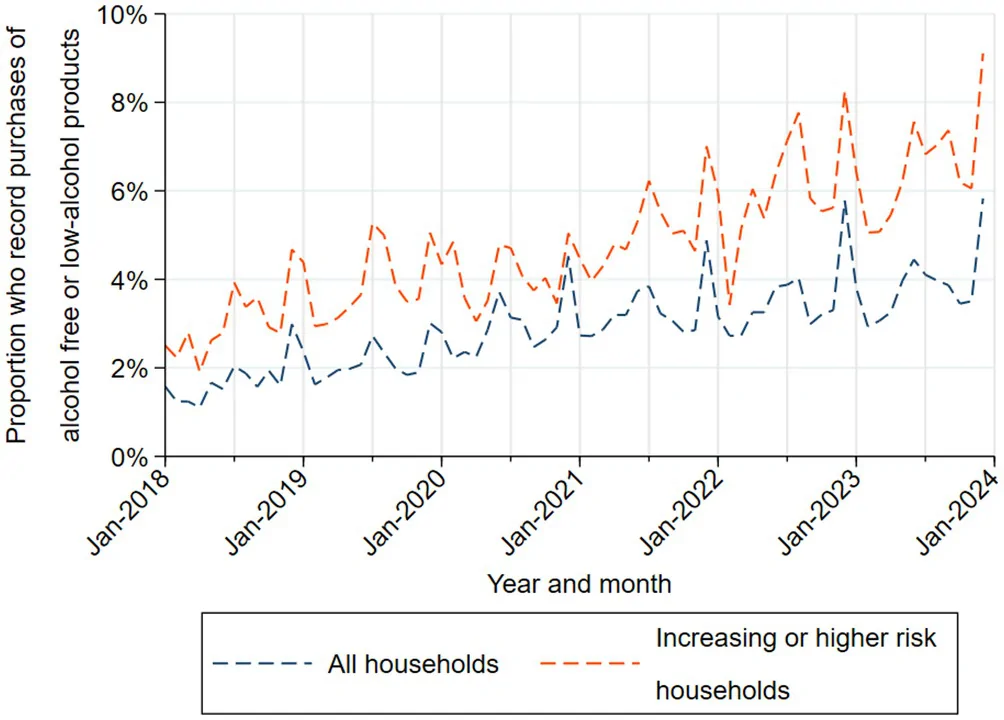
Monthly proportion of households recording purchases of alcohol‐free or low‐alcohol products (Worldpanel by Numerator Take Home data 2018–2023).

### 3.1 Primary Analyses

There were no significant associations between the prevalence of households purchasing no/lo products and our alcohol cut down outcomes: high motivation to cut down, current or past 12 month cut down attempts, most recent cut down attempt was a serious attempt or using no/lo drinks during most recent cut down attempt (Table 2; Figure 2). The confidence intervals for all four outcomes exclude clinically significant effects. There was a small uncertain *negative* association with current or past 12 month cut down attempts (*B*: −0.012 percentage points; 95% confidence interval −0.025 to 0.000). Full model specifications are reported in Table S1, auto‐correlation plots in Figure S1 and Granger causality tests in Table S3.

**TABLE 2:** Estimated associations between prevalence of households purchasing alcohol‐free or low‐alcohol products and alcohol cut down outcomes amongst at‐risk drinkers.^a^

Outcome measure (percentage points)	*B*	95% CI	*p*
High motivation to cut down			
Exposure^b^	−0.001	−0.013 to 0.011	0.879
Step change in April 2020	5.010	3.827 to 6.192	< 0.001
Current or past 12 month cut down attempts			
Exposure	−0.012	−0.025 to 0.000	0.056
Step change in April 2020	12.329	11.113 to 13.545	< 0.001
Most recent cut down attempt was a serious attempt^c^			
Exposure	0.004	−0.016 to 0.023	0.707
Step change in April 2020	4.965	1.345 to 8.585	0.007
Slope change from April 2020	0.281	0.052 to 0.510	0.016
Using alcohol‐free or low‐alcohol drinks during most recent cut down attempt^c^, ^d^			
Exposure	0.012	−0.023 to 0.046	0.507

Abbreviations: *B,* regression coefficient; CI, confidence interval.

^a^ At‐risk drinkers were defined as scoring ≥ 5 on the consumption questions or scoring ≥ 8 on all questions of the Alcohol Use Disorders Identification Test (AUDIT‐C screening test).

^b^ The proportion of households purchasing alcohol‐free or low‐alcohol products each month. This measure was log‐transformed and differenced to achieve stationarity, so coefficients and confidence intervals have been multiplied by log(1.01) to reflect the impact of a 1% change in prevalence compared to the previous month.

^c^ As a proportion of those who made an attempt to cut down in the last 12 months (including current attempts).

^d^ Time series starts in October 2020 and the model therefore does not include step or slope changes in April 2020.

**FIGURE 2:**
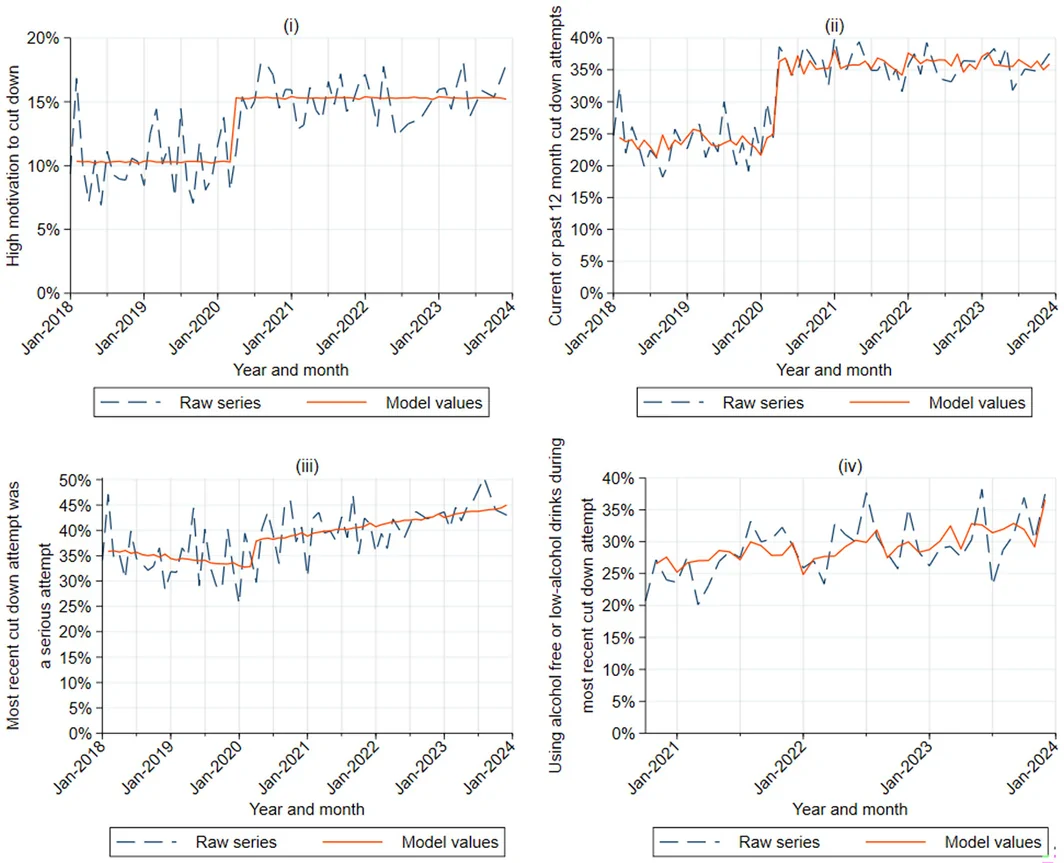
Monthly proportion of at‐risk drinkers who (i) report high motivation to cut down their alcohol consumption; (ii) report that they are either currently trying to restrict their alcohol consumption or have tried to restrict their alcohol consumption in the last 12 months; (iii) made a cut down attempt in the last 12 months and report that their most recent attempt to restrict their alcohol consumption was a serious attempt to cut down permanently; (iv) made a cut down attempt in the last 12 months and report that they used alcohol‐free or low‐alcohol drinks to help them try to cut down during their most recent attempt to restrict their alcohol consumption.

Our analysis incidentally found strong evidence that alcohol cut down outcomes were affected by the start of the COVID‐19 pandemic, operationalised as the start of April 2020 since we analysed monthly time series (Table 2; Figure 2). There was an immediate step change amongst at‐risk drinkers who reported a higher motivation to cut down, current or past 12 month cut down attempts, and that their most recent cut down attempt was a serious attempt. We also identified a change in the long‐term trend (from declining to increasing) in the proportion reporting that their most recent cut down attempt was a serious attempt.

### 3.2 Sensitivity Analyses

The pattern of results was consistent in sensitivity analyses where the exposure was the prevalence of increasing or higher risk households purchasing no/lo products (Table S2, Figure S2). All estimated effects of this exposure were also non‐significant, although there was more uncertainty with the association with current or past 12 month cut down attempts (*B*: −0.434 percentage points; 95% confidence interval −1.186 to 0.318).

## 4 Discussion

This study used time series analysis to evaluate the associations between the growing prevalence of households buying no/lo products and outcome measures relating to attempts to cut down alcohol consumption amongst at‐risk drinkers. Our descriptive results confirm previous evidence that no/lo products are a growing market, especially amongst households who are purchasing alcohol at increasing or higher risk levels [1]. We also show that no/lo products are also commonly—and increasingly—used by at‐risk drinkers to support their attempts to cut down alcohol consumption. However, the main time series analyses found no evidence that the prevalence of households buying no/lo products is associated with high levels of motivation to cut down, cut down attempts, serious cut down attempts or using no/lo products to support cut down attempts. We incidentally found evidence for a large impact of the COVID‐19 pandemic on increasing motivation to cut down and prevalence of cut down attempts, which is consistent with studies of the early pandemic period [28, 29], and that these changes have been sustained over time. These effects may have been driven by increased concern about alcohol consumption as people drinking at risky levels increased their alcohol consumption in the early COVID‐19 pandemic period [30]. However, the ATS also changed mode of administration during the pandemic, which may have at least partly driven the step effects seen [31].

This was the first study to examine the relationship between population‐level purchasing of no/lo products and attempts to cut down alcohol consumption. Strengths of this study include using time series methods that control for time‐varying confounders and data sources with large and nationally representative samples, which provide data on purchasing of no/lo products and alcohol cut down outcomes amongst at‐risk drinkers. A limitation is that the ATS is cross‐sectional, and people who previously drank at risky levels but reduced their consumption below this level were not asked to report on cut down attempts in the past year. These participants cannot be identified and, if the use of no/lo drinks to support cut down attempts leads to greater success, this could lead to an underestimate of the associations. However, the only existing individual‐level study on this topic found inconclusive evidence on whether using no/lo products to support cut down attempts was associated with successfully reducing alcohol consumption [32]. The Worldpanel data only captures no/lo purchases in the off‐trade. Off‐trade sales accounted for 90% of no/lo sold by volume in Great Britain in 2021, but it is possible there is a stronger relationship between on‐trade no/lo purchasing and cut down attempts [33].

If increasing prevalence of purchasing no/lo products is not leading to increased motivation to cut down, cut down attempts or increased use of no/lo products in cut down attempts amongst at‐risk drinkers, then policies supporting the availability and uptake of these products may not improve public health. People might primarily be drinking no/lo products in situations where they would already not have drunk alcohol. For example, they may use no/lo drinks as alternatives to other non‐alcoholic drinks when driving or during longer periods of abstinence (e.g., Dry January). Therefore, to reduce alcohol‐related harms, policy makers and public health actors should focus on well‐evidenced policy approaches such as the World Health Organization's ‘best buys’: increasing excise taxes on alcohol, bans or comprehensive restrictions on exposure to alcohol advertising and restrictions on physical availability of alcohol (such as by reduced hours of sale) [34].

Some unanswered questions remain on the use of no/lo products to support people to cut down their alcohol consumption. It may be useful to compare the evidence on no/lo products with the larger evidence base on e‐cigarette use to support quitting smoking (which includes evidence from different study designs to understand real‐world impacts) to identify areas for future research. Both no/lo products and e‐cigarettes are alternatives to harmful products that do not involve the primary harmful behaviour—drinking alcohol and inhaling combusted tobacco respectively. For e‐cigarettes, there is high certainty randomised controlled trial evidence that their use increases smoking quit rates [35]. Time series analysis has also shown that the rise in prevalence of smokers using e‐cigarettes is associated with increased success rate of quit attempts but found no clear association with quit attempt rate or overall quit rate [17]. This suggests that whilst e‐cigarettes have benefits when used to support quit attempts, wider use and availability of e‐cigarettes does not drive quit attempts. It is possible that no/lo drinks have a similar relationship with standard alcoholic drinks whereby their use to support cut down attempts improves success rates but increasing their use in the population overall does not lead to increased attempts to cut down alcohol consumption. However, as mentioned above, the only existing study on this topic was inconclusive [32]. A possible explanation for this is no/lo drinks lack the psychoactive component of alcoholic drinks (i.e., alcohol), whereas c‐cigarettes still contain nicotine. It is also possible that widespread use of no/lo drinks could be contributing to decreasing alcohol consumption outside of cut down attempts, amongst people drinking at moderate levels, in particular subgroups of the population that our study lacked power to examine, or that the no/lo market is not yet large enough to detect associations with cut down attempts. There may be a threshold prevalence of no/lo drink use where effects become substantial. Future research on these questions would further our understanding of the relationship between the use of no/lo products and standard alcohol consumption.

## 5 Conclusions

There was no clear association between the rise in household prevalence of purchasing alcohol‐free and low‐alcohol products in England and increased motivation to cut down alcohol consumption, attempts to cut down alcohol consumption or use of no/lo products to support cutting down amongst at‐risk drinkers. The evidence base on alcohol‐free and low‐alcohol products remains uncertain. However, to date it is not consistent with promoting the availability of these products as a viable alternative to well‐evidenced alcohol policy options such as increasing alcohol prices, reducing exposure to alcohol marketing and restricting the physical availability of alcohol [34].

## Author Contributions

Each author certifies that their contribution to this work meets the standards of the International Committee of Medical Journal Editors.

## Funding

This study was funded by the National Institute for Health and Care Research (NIHR) Public Health Research program (NIHR135310). A.K.S. is funded by the NIHR School for Public Health Research (grant ref.: NIHR204000). The views expressed are those of the author(s) and not necessarily those of the NIHR or the Department of Health and Social Care. The data collection is also funded by Cancer Research UK (grant ref.: PRCRPG‐Nov21\100002) in England and the UK Prevention Research Partnership (via SPECTRUM, grant ref.: MR/S037519/1) in Scotland and Wales.

## Conflicts of Interest

All data used in this paper were purchased under licence using funding from NIHR135310 (see funding). Use of the Worldpanel dataset is allowed under the terms of the contract and nondisclosure agreement between Worldpanel by Numerator (formerly Kantar) and the University of Sheffield, which requires research outputs to be submitted to the data provider ahead of publication. The data provider's right to request changes is limited to matters of accuracy regarding the data. The data provider played no further role in the research process, including in conception, design, analysis, interpretation, write‐up or the decision to publish. I.K., J.B. and J.H. have received funding from Alcohol Change UK, including for a separate ongoing research project on alcohol‐free and low‐alcohol drinks that began in January 2025. Since beginning that project, the authors have become aware that Alcohol Change UK received < 0.6% of its funds in 2024–2025 from Lucky Saint, an organiZation that produces and sells alcohol‐free drinks and owns a pub that sells standard alcoholic drinks. In March 2025, Lucky Saint became an associate member of The Portman Group.

## Supporting information


**Table S1:** Estimated associations between the prevalence of purchasing alcohol‐free and low‐alcohol products and alcohol‐related outcomes amongst people who drink at hazardous or harmful levels, by model specification.
**Table S2:** Estimated associations between the prevalence of increasing or higher risk households purchasing alcohol‐free and low‐alcohol products and alcohol‐related outcomes amongst people who drink at hazardous or harmful levels: sensitivity analysis.
**Figure S1:** Auto‐correlation (a) and partial auto‐correlation (b) plots for residuals of models estimating the associations between prevalence of households purchasing alcohol‐free or low‐alcohol products and alcohol cut down outcomes amongst at‐risk drinkers. Outcomes are high motivation to cut down their alcohol consumption (1), either currently trying to restrict their alcohol consumption or have tried to restrict their alcohol consumption in the last 12 months (2), made a cut down attempt in the last 12 months and report that their most recent attempt to restrict their alcohol consumption was a serious attempt to cut down permanently (3), and made a cut down attempt in the last 12 months and report that they used alcohol‐free or low‐alcohol drinks to help them try to cut down during their most recent attempt to restrict their alcohol consumption (4).
**Figure S2:** Auto‐correlation (a) and partial auto‐correlation (b) plots for residuals of models estimating the associations between the proportion of increasing or higher risk households who record purchases of alcohol‐free or low‐alcohol products in each month and alcohol cut down outcomes amongst at‐risk drinkers. Outcomes are high motivation to cut down their alcohol consumption (1), either currently trying to restrict their alcohol consumption or have tried to restrict their alcohol consumption in the last 12 months (2), and made a cut down attempt in the last 12 months and report that their most recent attempt to restrict their alcohol consumption was a serious attempt to cut down permanently (3).
**Table S3:** Estimated associations between the prevalence of purchasing alcohol‐free and low‐alcohol products and alcohol‐related outcomes amongst people who drink at hazardous or harmful levels: results for Wald tests of Granger causality.

## Data Availability

Data will not be shared. Data used in these analyses are a commercial product licensed for use by the University of Sheffield and cannot be shared. The data are available for in‐person inspection at the University of Sheffield by researchers on request.
