# Supplementary material for: Are Increases in Purchasing Alcohol‐Free and Low‐Alcohol Drinks Associated With Attempts to Cut Down on Alcohol Consumption in England? A Time Series Analysis
Source: Drug Alcohol Rev. 2026 Oct 9;45(7):e70278. doi: 10.1111/dar.70278 (PMC13651000; doi:10.1111/dar.70278)
Supplement: Supplementary file 1 [file DAR-45-0-s001.docx]

Table S1. Estimated associations between prevalence of purchasing alcohol free and low-alcohol products and alcohol-related outcomes among people who drink at hazardous or harmful levels^1^, model specifications

| Outcome measure | Seasonal terms | Auto-regressive terms | Integrating terms | Moving average terms | Trend terms | Portmanteau test p-value |
| --- | --- | --- | --- | --- | --- | --- |
| Primary analysis - Associations between the proportion of households who record purchases of alcohol free or low-alcohol products in each month and the proportion of people drinking at hazardous or harmful levels who report: | | | | | | |
| High motivation to cut down | 0,0,0 | 0 | 0 | 0 | None | 0.518 |
| Current or past 12 month cut down attempts | 0,0,0 | 6 | 0 | 0 | None | 0.664 |
| Most recent cut down attempt was a serious attempt | 0,0,0 | 0 | 0 | 0 | Linear | 0.957 |
| Using alcohol free or low-alcohol drinks during most recent cut down attempt | 0,0,0 | 5 | 0 | 0 | Linear | 0.878 |
| Sensitivity analysis – Associations between the proportion of increasing or higher risk households^2^ who record purchases of alcohol free or low-alcohol products in each month and the proportion of people drinking at hazardous or harmful levels who report: | | | | | | |
| High motivation to cut down | 0,0,0 | 0 | 0 | 0 | None | 0.543 |
| Current or past 12 month cut down attempts | 0,0,0 | 6 | 0 | 0 | None | 0.529 |
| Most recent cut down attempt was a serious attempt | 0,0,0 | 0 | 0 | 0 | Linear | 0.976 |
| Using alcohol free or low-alcohol drinks during most recent cut down attempt | NA^3^ |  |  |  |  |  |

^1^ Drinking at hazardous or harmful levels was defined as a score of ≥ 5 on the consumption questions of the Alcohol Use Disorders Identification Test (AUDIT-C screening test). ^2^ Increasing or higher risk households were defined as purchasing more than 14 units (1 UK unit = 10ml pure alcohol) of alcohol per adult in the household per week on average that month. ^3^ This model was not fit due to evidence of reverse causality between our exposures and outcomes, as per our protocol (see Table S3).

Table S2. Estimated associations between prevalence of increasing or higher risk households^1^ purchasing alcohol free and low-alcohol products and alcohol-related outcomes among people who drink at hazardous or harmful levels^2^, sensitivity analysis

| Outcome measure | B | 95% Confidence interval | p-value |
| --- | --- | --- | --- |
| High motivation to cut down | | | |
| Exposure^3^ | 0.217 | -0.366, 0.810 | 0.474 |
| Step change in April 2020 | 4.987 | 3.814, 6.160 | <0.001 |
| Current or past 12 month cut down attempts | | | |
| Exposure | -0.434 | -1.186, 0.318 | 0.258 |
| Step change in April 2020 | 12.340 | 11.111, 13.569 | <0.001 |
| Most recent cut down attempt was a serious attempt | | | |
| Exposure | 0.531 | -0.814, 1.875 | 0.439 |
| Step change in April 2020 | 4.925 | 1.283, 8.566 | 0.008 |
| Slope change from April 2020 | 0.273 | 0.043, 0.502 | 0.020 |
| Using alcohol free or low-alcohol drinks during most recent cut down attempt | | | |
| Exposure | NA^4^ |  |  |

B = Regression coefficient. ^1^ Increasing or higher risk households were defined as purchasing more than 14 units (1 UK unit = 10ml pure alcohol) of alcohol per adult in the household per week on average that month. ^2^ Drinking at hazardous or harmful levels was defined as a score of ≥ 5 on the consumption questions of the Alcohol Use Disorders Identification Test (AUDIT-C screening test). ^3^ The proportion of increasing or higher risk households purchasing alcohol free or low-alcohol products each month. This measure was differenced to achieve stationarity, so coefficients reflect the impact of a 1% change in prevalence compared to the previous month. ^4^ This model was not fit due to evidence of reverse causality between our exposures and outcomes, as per our protocol (see Table S3).

^1^ At-risk drinkers were defined as scoring ≥ 5 on the consumption questions or scoring ≥ 8 on all questions of the Alcohol Use Disorders Identification Test (AUDIT-C screening test).

Figure S1. Auto-correlation (a) and partial auto-correlation (b) plots for residuals of models estimating the associations between prevalence of households purchasing alcohol-free or low-alcohol products and alcohol cut down outcomes among at-risk drinkers^1^. Outcomes are high motivation to cut down their alcohol consumption (1), either currently trying to restrict their alcohol consumption or have tried to restrict their alcohol consumption in the last 12 months (2), made a cut down attempt in the last 12 months and report that their most recent attempt to restrict their alcohol consumption was a serious attempt to cut down permanently (3), and made a cut down attempt in the last 12 months and report that they used alcohol-free or low-alcohol drinks to help them try to cut down during their most recent attempt to restrict their alcohol consumption (4).

^1^ Increasing or higher risk households were defined as purchasing more than 14 units (1 UK unit = 10ml pure alcohol) of alcohol per adult in the household per week on average that month. ^2^ At-risk drinkers were defined as scoring ≥ 5 on the consumption questions or scoring ≥ 8 on all questions of the Alcohol Use Disorders Identification Test (AUDIT-C screening test).

Figure S2. Auto-correlation (a) and partial auto-correlation (b) plots for residuals of models estimating the associations between the proportion of increasing or higher risk households^1^ who record purchases of alcohol free or low-alcohol products in each month and alcohol cut down outcomes among at-risk drinkers^2^. Outcomes are high motivation to cut down their alcohol consumption (1), either currently trying to restrict their alcohol consumption or have tried to restrict their alcohol consumption in the last 12 months (2), and made a cut down attempt in the last 12 months and report that their most recent attempt to restrict their alcohol consumption was a serious attempt to cut down permanently (3).

Table S3. Estimated associations between prevalence of purchasing alcohol free and low-alcohol products and alcohol-related outcomes among people who drink at hazardous or harmful levels^1^, results for Wald tests of Granger causality

|  | | Causality | | Reverse causality | |
| --- | --- | --- | --- | --- | --- |
| Outcome measure | Number of lags tested | chi-squared | p-value | chi-squared | p-value |
| Primary analysis - Associations between the proportion of households who record purchases of alcohol free or low-alcohol products in each month and the proportion of people drinking at hazardous or harmful levels who report: | | | | | |
| High motivation to cut down | 7 | 28.59 | <0.001 | 9.93 | 0.193 |
| Current or past 12 month cut down attempts | 7 | 13.21 | 0.067 | 11.67 | 0.112 |
| Most recent cut down attempt was a serious attempt | 4 | 4.47 | 0.346 | 5.94 | 0.204 |
| Using alcohol free or low-alcohol drinks during most recent cut down attempt | 6 | 14.95 | 0.021 | 6.45 | 0.374 |
| Sensitivity analysis – Associations between the proportion of increasing or higher risk households^2^ who record purchases of alcohol free or low-alcohol products in each month and the proportion of people drinking at hazardous or harmful levels who report: | | | | | |
| High motivation to cut down | 7 | 11.69 | 0.111 | 7.82 | 0.347 |
| Current or past 12 month cut down attempts | 7 | 11.48 | 0.119 | 10.83 | 0.146 |
| Most recent cut down attempt was a serious attempt | 2 | 9.33 | 0.009 | 3.98 | 0.137 |
| Using alcohol free or low-alcohol drinks during most recent cut down attempt | 5 | 12.78 | 0.026 | 14.13 | 0.015 |

^1^ Drinking at hazardous or harmful levels was defined as a score of ≥ 5 on the consumption questions of the Alcohol Use Disorders Identification Test (AUDIT-C screening test). ^2^ Increasing or higher risk households were defined as purchasing more than 14 units (1 UK unit = 10ml pure alcohol) of alcohol per adult in the household per week on average that month.
